# Magnetodielectric and Rheological Effects in Magnetorheological Suspensions Based on Lard, Gelatin and Carbonyl Iron Microparticles

**DOI:** 10.3390/ma17163941

**Published:** 2024-08-08

**Authors:** Octavian Madalin Bunoiu, Ioan Bica, Eugen Mircea Anitas, Larisa Marina Elisabeth Chirigiu

**Affiliations:** 1 Department of Physics, West University of Timisoara, V. Parvan Avenue 4, 300223 Timisoara, Romania; madalin.bunoiu@e-uvt.ro; 2 Department of Physics, Craiova University, A. I. Cuza Street 13, 200585 Craiova, Romania; 3 Joint Institute for Nuclear Research, Joliot-Curie 6, 141980 Dubna, Russia; anitas@theor.jinr.ru; 4 Horia Hulubei National Institute of Physics and Nuclear Engineering, 077125 Magurele, Romania; 5 Faculty of Pharmacy, University of Medicine and Pharmacy Craiova, Petru Rareș 2, 200349 Craiova, Romania; larisa.chirigiu@umfcv.ro

**Keywords:** lard, gelatine, carbonyl iron microparticles, viscosity, relative dielectric permittivity, dielectric loss factor, magnetodielectric effect

## Abstract

This study aims to develop low-cost, eco-friendly, and circular economy-compliant composite materials by creating three types of magnetorheological suspensions (MRSs) utilizing lard, carbonyl iron (CI) microparticles, and varying quantities of gelatin particles (GP). These MRSs serve as dielectric materials in cylindrical cells used to fabricate electric capacitors. The equivalent electrical capacitance (*C*) of these capacitors is measured under different magnetic flux densities (B≤160 mT) superimposed on a medium-frequency electric field (*f* = 1 kHz) over a period of 120 s. The results indicate that at high values of *B*, increasing the GP content to 20 vol.% decreases the capacitance *C* up to about one order of magnitude compared to MRS without GP. From the measured data, the average values of capacitance $C_{m}$ are derived, enabling the calculation of relative dielectric permittivities ($\epsilon_{r}^{\prime }$) and the dynamic viscosities (η) of the MRSs. It is demonstrated that $\epsilon_{r}^{\prime }$ and η can be adjusted by modifying the MRS composition and fine-tuned through the magnetic flux density *B*. A theoretical model based on the theory of dipolar approximations is used to show that $\epsilon_{r}^{\prime }$, η, and the magnetodielectric effect can be coarsely adjusted through the composition of MRSs and finely adjusted through the values *B* of the magnetic flux density. The ability to fine-tune these properties highlights the versatility of these materials, making them suitable for applications in various industries, including electronics, automotive, and aerospace.

## 1. Introduction

Magnetorheological suspensions (MRSs) consist of a liquid matrix in which magnetizable microparticles and additives are dispersed [1,2,3,4,5,6]. When exposed to a magnetic field [1,2,3], the magnetizable microparticles transform into magnetic dipoles. These dipoles interact, forming aggregates in the shape of columns. The strength of these columns is determined by the magnetic flux density and the magnetic properties of the magnetizable phase [2]. The formation of these aggregates results in significant changes to the viscosity [1], electrical conductivity [7], and dielectric properties [4] of MRSs. These effects are beneficial for applications in artificial intelligence [5,8], vibration dampers [6], clutches [9], or passive electrical circuit elements [7].

Traditionally, MRSs often utilize synthetic oils, such as paraffin, as liquid matrix and high-purity additives, which can be expensive and environmentally harmful [10,11,12]. Synthetic oils and certain polymeric additives do not decompose easily, contributing to long-term environmental pollution. This may hamper widespread adoption of MRS technology, particularly in cost-sensitive applications. Therefore, in the last years, preparation of MRSs with environmentally friendly and recyclable components has been an active research area [1,13,14,15,16]. These include MRSs based on honey with carbonyl iron (CI) microparticles [17] and turmeric powder [13], composites based on honey with CI microparticles and beeswax [14], or MRSs based on nanocellulose [1], nanolignocelluloses [15], and gelatine-coated CI microparticles [16].

An essential requirement for MRSs is the ability to precisely control their viscosity. This is a key feature for their application in devices such as dampers and clutches [18,19]. However, achieving stable and tunable viscosity in eco-friendly and low-cost MRSs remains a challenge. The dielectric properties of MRSs, such as relative dielectric permittivity and dielectric loss factor, are also critical for applications in capacitors and other electronic components [20,21]. Ensuring that these properties can be finely tuned and remain stable under varying operational conditions is a significant challenge. In addition, sedimentation of magnetizable particles is a common issue that affects the long-term stability and performance of the suspension [11,22,23]. Particles tend to settle over time due to gravity and other contributing factors such as particle-particle interactions, oxidation of the magnetizable particles, Brownian motion, and the viscosity of the base fluid, leading to a non-uniform distribution and inconsistent magnetic and rheological properties.

By addressing the challenges of cost, environmental impact, viscosity control, dielectric properties, and sedimentation, the present work utilizes lard [24,25], animal gelatin particles (GP; [26]), and CI microparticles to develop a sustainable alternative for traditional MRS. Lard is a promising candidate due to its cost-effectiveness, biodegradability, and renewability. Lard’s high viscosity at room temperature helps in preventing the sedimentation of GP and CI microparticles. This ensures a more uniform distribution of particles, maintaining consistent magnetic and rheological properties over time. It is used in the production of biodiesel-type fuels [27], antioxidants [28], has beneficial effects on the intestinal microbiome [29], and can be processed into glycerides and hydrogenated glycerides for cosmetic products [30]. Animal gelatin, a fibrous protein derived from the tissues of pigs and cattle [31], finds applications across various industries, including food, pharmaceuticals, and tissue regeneration. Its ability to mold easily and form films with micrometric dimensions makes it suitable for use in MRSs.

This study aims to demonstrate that suspensions based on lard, GP, and CI microparticles exhibit dielectric and magnetodielectric properties similar to those of traditional MRSs [1,2,3,4,5,6]. Thus, the suspensions are used as dielectric materials in cylindrical cells for the fabrication of electric capacitors. The electric capacitance and resistance of these capacitors are measured under different magnetic flux densities (from 0 to 160 mT) and a medium-frequency electric field (1 kHz) over a period of 120 s. Further, using the model of dipolar approximation [32,33,34,35], the dynamic viscosity, relative dielectric permittivity, and magnetodielectric effect are investigated as a function of magnetic flux density and the ratio of the volume fraction of lard to GP. This model allows adjustments through the composition of the MRSs and variations in the magnetic flux density by considering the interactions of magnetic dipoles within the suspensions.

To address these issues, this paper is organized as follows: Section 2 details the materials and methods used in the preparation of the MRSs, dielectric permittivity, and other key properties of the components. Section 3 describes the fabrication process of planar electrical capacitors (PECs) utilizing the prepared MRSs as dielectric materials. In Section 4, we present the experimental setup and the methodology for measuring the electrical properties of the PECs under various magnetic flux densities. Section 5 presents the theoretical background. Section 6 discusses the results of these measurements, focusing on the stability and performance of the PECs with different compositions of lard, GP, and CI microparticles. Section 7 provides a detailed discussion of the findings in the context of existing literature and theoretical models, highlighting the implications and potential applications of the developed MRSs. Finally, Section 8 concludes the paper by summarizing the key contributions and suggesting directions for future research.

## 2. Preparation of MRSs

### 2.1. Materials

**Figure 1:**
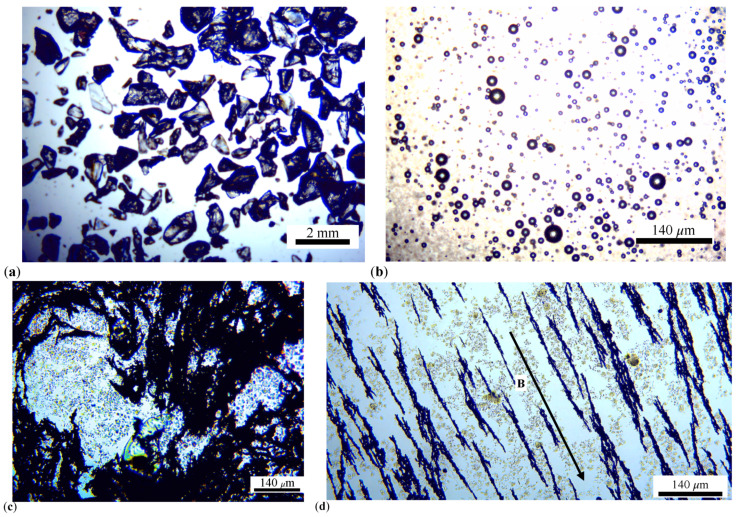
Photographs taken with the OPTIKA microscope (made in Italy): (**a**) Bulk GPs. (**b**) Field of GPs (dark spots) dispersed in a lard film; (**c**) MRS without a magnetic field. (**d**) MRS in a magnetic field with a magnetic flux density of approximately 50 mT. In (**c**,**d**) black regions represent CI microparticles and gray-like particles represent GPs dispersed in lard.

The materials used for producing MRS are as follows:

- Lard, produced by Elit (Alba Iulia, Romania), supplied through commercial stores.
- Animal gelatin, from Dr. Oetker SRL (Curtea de Arges, Romania), supplied through grocery stores. The gelatin is in the form of white granules (GP) with equivalent diameters less than or equal to 1 mm as shown in Figure 1a.
- CI microparticles, are produced by Sigma-Aldrich (St. Louis, MO, USA). Their sizes are between 4.5 μm and 5.4 μm.

The relative dielectric permittivity ($\varepsilon_{r}^{\prime }$) and dielectric loss factor ($\varepsilon_{r}^{\prime \prime }$) are measured a frequency of f=1 kHz. The values of measured mass densities (ρ), $\varepsilon_{r}^{\prime }$ and $\varepsilon_{r}^{\prime \prime }$, are listed in Table 1 together with the loss tangent $D=\varepsilon_{r}^{\prime \prime }/\varepsilon_{r}^{\prime }$.

### 2.2. Method

The manufacturing of MRS suspensions is carried out through the following steps:

1. The volume $V_{\mathrm{lard}}$ of lard, $V_{\mathrm{CI}}$ of CI microparticles, and $V_{\mathrm{GP}}$ of GP are measured. The corresponding values are listed in Table 2.
2. In a Berzelius beaker, the volumes $V_{\mathrm{lard}}$ and $V_{\mathrm{CI}}$ corresponding to ${\mathrm{MRS}}_{1}$ from Table 2 are introduced. The components, consisting of lard and CI microparticles, are mixed while heating (approximately at 250 °C) for about five minutes. The mixing continues until the liquid mixture reaches ambient temperature (approximately 27 °C). At the end of this stage, a dark-colored mixture, hereafter referred to as ${\mathrm{MRS}}_{1}$ suspension, is obtained.
3. Volumes of 3.2 ${\mathrm{cm}}^{3}$ of lard and 0.4 ${\mathrm{cm}}^{3}$ of GP are measured and introduced into a Berzelius beaker. In a second Berzelius beaker, are introduced 2.8 ${\mathrm{cm}}^{3}$ of lard and 0.8 ${\mathrm{cm}}^{3}$ of GP. The mixtures in the Berzelius beakers are homogenized by turn at a temperature of approximately 250 °C for about five minutes, after which the mixing continues until the liquid mixtures reach ambient temperature (approximately 27 °C). A film of the prepared mixture is deposited on a glass slide. The resulting image is shown in Figure 1b. It can be observed from this figure that the formed microparticles have micrometric dimensions with an average diameter of 6.94 ± 0.55 μm (see Appendix A for details) and have a spherical shape.
4. In the Berzelius beaker with 3.2 ${\mathrm{cm}}^{3}$ of lard and 0.4 ${\mathrm{cm}}^{3}$ of GP, are introduced 0.4 ${\mathrm{cm}}^{3}$ of CI microparticles, and the mixture is heated to approximately 150 °C for about five minutes. At the end of this period, the mixture is further homogenized until it reaches ambient temperature. At the end of this stage, the ${\mathrm{MRS}}_{2}$ suspension is formed.
5. In the Berzelius beaker with 2.8 ${\mathrm{cm}}^{3}$ of lard and 0.8 ${\mathrm{cm}}^{3}$ of GP, 0.4 ${\mathrm{cm}}^{3}$ of CI microparticles are introduced, and the mixture is heated to approximately 150 °C for about five minutes. At the end of this period, the mixture further homogenizes until it reaches ambient temperature. At the end of this stage, the ${\mathrm{MRS}}_{3}$ suspension is formed.

The MRS suspensions thus prepared have volume fractions $\Phi_{\mathrm{lard}}$, $\Phi_{\mathrm{CI}}$, and $\Phi_{\mathrm{GP}}$ with values specified in Table 2. In the study of the magnetic properties of composite materials, the relationship $\mu_{0}\sigma_{s_{\mathrm{MRS}}}=\mu_{0}\sigma_{m_{\mathrm{CI}}}\Phi_{\mathrm{CI}}$ [36] is used to determine their specific saturation magnetization $\sigma_{s_{\mathrm{MRS}}}$, where $\mu_{0}$ is the magnetic constant of the vacuum, $\Phi_{\mathrm{CI}}$ is the volume fraction of CI microparticles, and $\sigma_{\mathrm{mCI}}$ is the specific saturation magnetization of the CI microparticles. For $\sigma_{m_{\mathrm{CI}}}=218$ ${\mathrm{Am}}^{2}$/kg [37,38] and $\Phi_{\mathrm{CI}}=10$ vol.% introduced into the specified relation, the value $\sigma_{s_{\mathrm{MRS}}}=21.8$ ${\mathrm{Am}}^{2}$/kg is obtained. A film of the ${\mathrm{MRS}}_{3}$ suspension is visualized using an Optika microscope (Figure 1c). Upon applying a magnetic field (Figure 1d), the CI microparticles form chains of magnetic dipoles along the direction of B, through the field formed by the GP and lard microparticles.

## 3. Fabrication of PECs

### 3.1. Materials for PECs

The materials needed for manufacturing PECs are:

1. Laminated board (LB) based on epoxy resin, reinforced with fiberglass, with one side plated with copper, has a thickness of 0.35 μm. The LB is obtained from HobbyMarket (Bucuresti, Romania) and is delivered in dimensions of 210 mm × 100 mm × 1.5 mm.
2. Non-slip rubber pad (RP), type CAR-BOY (made in Japan) and supplied by Hornbach (Timisoara, Romania). The RP pad has a diameter of 40 mm and a thickness of 2 mm.
3. Surgical adhesive tape Durapore (ST), manufactured by 3M EMEA GmbH (Langenthal, Switzerland), and supplied through Help Net (Bucuresti, Romania). The tape is 5 cm wide and 9 m long.

### 3.2. Method for Obtaining PECs

**Figure 2:**
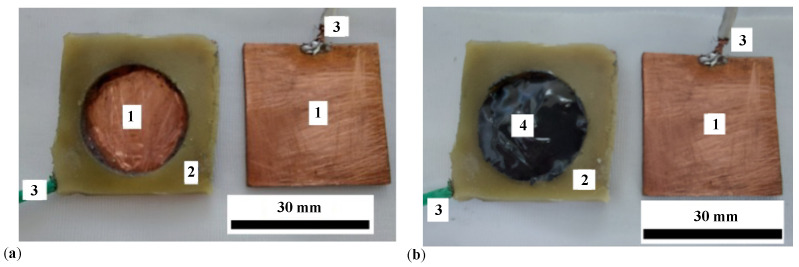
(**a**) MC with attached LB. (**b**) Measurement cell with MRS and attached LB. 1—copper foil of the LB; 2—ring made from the RP pad; 3—flexible electric conductor; 4—MRS.

**Figure 3:**
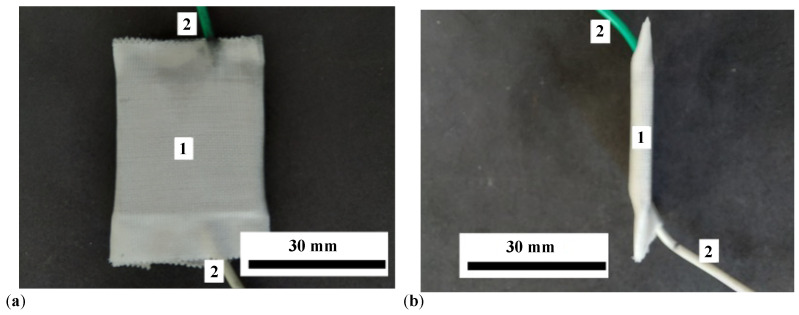
Images of PEC. (**a**) Front view. (**b**) Side view. 1—PEC body consolidated with ST tape, 2—flexible electric conductors.

**Figure A2:**
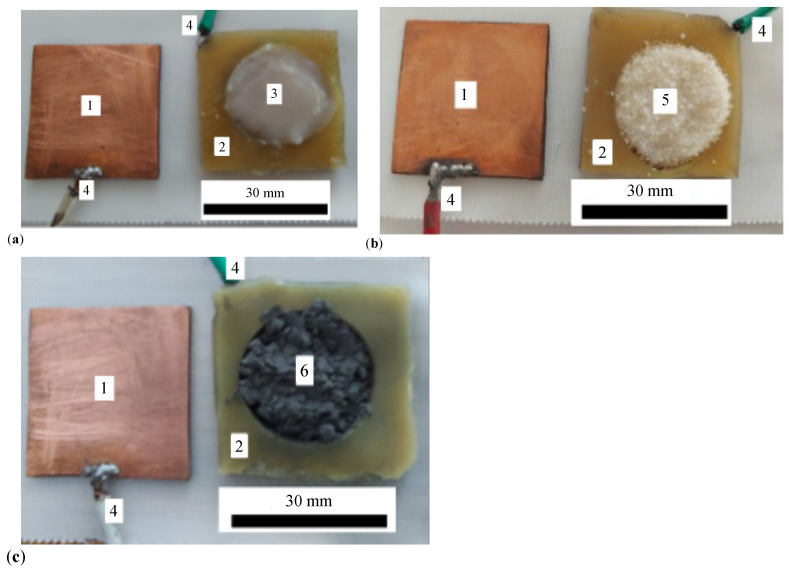
Measuring cells with lard (**a**), GP (**b**) and CI microparticles (**c**). 1—copper electrode, 2—insulating ring with a diameter of 20 mm and thickness of 2 mm, 3—lard, 4—copper conductor, 5—GP, 6—CI microparticles.

The main steps in preparing PECs are:

1. LB is cut into six pieces. Each piece has dimensions of 30 mm × 30 mm × 1.5 mm.
2. Three rings with an inner diameter of 20 mm are cut from the RP pad.
3. On a batch of three LBs, an adhesive pad is fixed on top of each one. At the end of this stage, three measurement cells (MCs) are obtained, each with an attached LB, as shown in (Figure 2a). An MC with MRS inside is shown in Figure 2b.
4. On top of the MC filled with MRS (Figure 2b), the copper-coated side of the LB is fixed by pressing. The assembly thus realized is consolidated with ST tape. At the end of this stage, three capacitors denoted by ${\mathrm{PEC}}_{1}$, ${\mathrm{PEC}}_{2}$, and ${\mathrm{PEC}}_{3}$ are obtained, as shown in Figure 3 (see details in Figure A2 in Appendix B).

**Figure 4:**
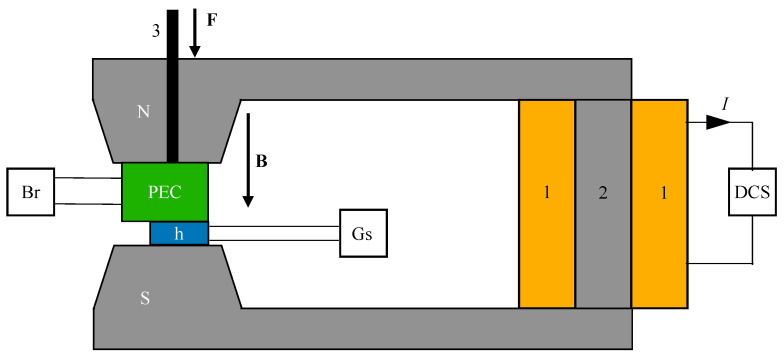
Experimental setup. DCS—direct current source; Br—RLC bridge; Gs—gaussmeter; h—Hall probe; PEC—electric capacitor; N and S—magnetic poles; **B**—magnetic flux density vector; *I*—electric current intensity through the electromagnet coil; 1—coil; 2—magnetic yoke; 3—non-magnetic axis; **F**—compressive force vector.

The experimental setup for studying MRSs has the overall configuration shown in Figure 4. The setup includes an in-house-built electromagnet composed of a magnetic yoke (position 1) and a coil (position 2) connected to the DCS source. Between the magnetic poles N and S, the PEC capacitor and the Hall probe (h) of the gaussmeter (Gs) are mechanically fixed via the non-magnetic axis (position 3). The PEC capacitors are connected to the RLC bridge (Br).

By adjusting the current intensity *I* through the coil up to a maximum of 5 $A_{\mathrm{dc}}$, the magnetic flux density *B* between the magnetic poles N and S can be continuously adjusted up to a maximum of 400 mT. The DCS source, model RXN-3020D, is from Shenzhen Ever Good Electronic Co., Ltd. (Shenzhen, China). The *B* values of the magnetic flux density are measured with the gaussmeter Gs type DX-102 and the Hall probe h. The gaussmeter and Hall probe h are from Dexing-Magnetic Industrial Park (Xiamen, China). The RLC bridge (Br; CHY 41R type) is from Firemate (Tainan, Taiwan). During measurements, the bridge is connected in parallel mode and at a frequency of f=1 kHz. This is a standard frequency for medium-frequency electric field measurements, relevant to practical applications, and technically feasible with high accuracy and precision using standard laboratory equipment.

## 4. Measurements of Electrical Properties

Between the N and S poles of the electromagnet in Figure 4, we introduce by turn the capacitors ${\mathrm{PEC}}_{1}$, ${\mathrm{PEC}}_{2}$, and ${\mathrm{PEC}}_{3}$, along with the Hall probe h, securing them mechanically. The capacitors are subjected to a mechanical pressure of approximately 9kPa, applied by an 800g lead mass. Each capacitor is electrically connected to the RLC bridge, set on the *C* mode for measuring electrical capacitance. The ambient temperature is $27\pm {0.5\;}^{\circ }C$. Through the RS232C interface of the RLC bridge, the capacitance values measured in the magnetic field at the initial moment (t=0 s) and at t=120 s are recorded by a computing unit not shown in Figure 4. During the measurements, the *B* values of the magnetic flux density are increased in steps of 10 mT, up to a maximum of 160 mT.

## 5. Theoretical Background

**Figure 5:**
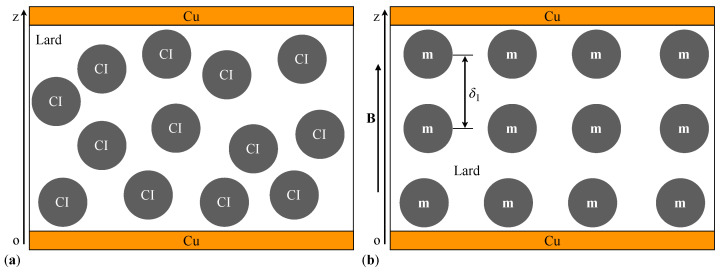
Cross-section through capacitors with a dielectric composed of lard and CI microparticles (model) under: (**a**) absence of a magnetic field; (**b**) presence of a magnetic field. Cu—copper foil, **m**—magnetic moment vector, **B**—magnetic flux density vector, Oz—coordinate axis.

**Figure 6:**
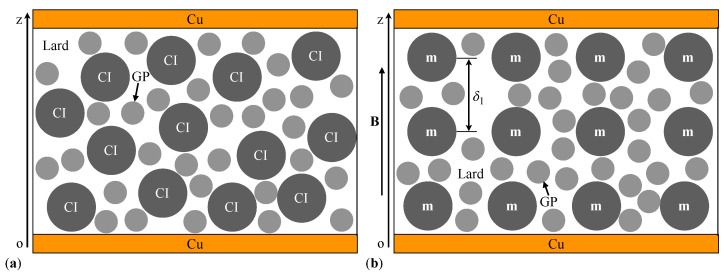
Cross-section through capacitors with a dielectric composed of lard, GP, and CI microparticles (model) under: (**a**) absence of a magnetic field; (**b**) presence of a magnetic field. The symbols are the same as above.

For the obtained PECs (see Figure 3), we model the dielectric material without and with GP, as shown in Figure 5 and Figure 6, respectively. We consider that the CI microparticles in these figures are spherical and have a diameter equal to the average diameter, $d_{m}\approx 5\;\mu m$. In a magnetic field, the CI microparticles magnetize instantaneously, forming magnetic dipoles. The dipoles **m** align in the direction of **B**, parallel to the Oz coordinate axis. At the moment of applying **B**, considered the initial moment ($t_{0}=0\;s$), the distance between two neighboring dipoles **m** is approximated by the relation [39]:

(1) $$\delta_{1}=\frac{d_{m}}{\sqrt[{3}]{\Phi_{\mathrm{CI}}}}\approx 10.77\;\mu m,\;\mathrm{for}\;\mathrm{the}\;\mathrm{suspension}\;{\mathrm{MRS}}_{1},$$

and by the relation:

(2) $$\delta_{i}=\frac{d_{m}}{\sqrt[{3}]{\frac{\Phi_{\mathrm{CI}}}{1+\Phi_{\mathrm{GP}}}}}\approx \left\{\begin{array}{cc} 11.12\;\mu m, & \mathrm{for}\;\mathrm{the}\;\mathrm{suspension}\;{\mathrm{MRS}}_{2}\\ 11.45\;\mu m, & \mathrm{for}\;\mathrm{the}\;\mathrm{suspension}\;{\mathrm{MRS}}_{3} \end{array}\right.,$$

with i=2,3. Here $d_{m}$ and $\Phi_{\mathrm{CI}}$ are the average diameter and volume fraction of the CI microparticles, and $\Phi_{\mathrm{GP}}$ is the volume fraction of GP.

The dipole magnetic moment projected on the Oz coordinate axis is calculated with the expression [39]:

(3) $$m=\frac{\pi }{2}d_{m}^{2}\frac{B}{\mu_{0}},$$

where $\mu_{0}$ is the magnetic constant of the vacuum. Between the dipoles **m** (see Figure 5a and Figure 6a), along the Oz axis, magnetic interactions of intensity occur [39]:

(4) $$f_{m_{z}}=\frac{3\mu_{0}m^{2}}{4z^{4}},$$

where *m* is the magnitude of the dipole moment and *z* is the distance between the centers of mass of the dipoles **m** at a moment $t>t_{0}$. From Equations (3) and (4); for $z=d_{m}$, we obtain:

(5) $$f_{m_{z}}=-\frac{3\pi d_{m}^{2}B^{2}}{4\mu_{0}}.$$

The negative sign in this expression indicates that the dipoles **m** in the chain attract each other. In the time interval dt, the dipoles **m** in each chain approach by a distance $dz_{i}$ (i=1,2,3). The movement of the dipoles **m** is opposed by the resistance force $f_{r_{z}}$ of the lard. The magnitude of $f_{r_{z}}$ is calculated with the relation [39]:

(6) $$f_{r_{z}i}=3\pi d_{m}\eta_{i}\frac{dz_{i}}{dt},\;\mathrm{with}\;i=1,2,3,$$

where $\eta_{i}$ is the viscosity of the medium in which it takes place the movement of dipoles **m**.

By considering that the mass of CI used is very small, at an arbitrary moment *t*, between the quantities $f_{m_{z}}$ and $f_{r_{z_{i}}}$ (with i=1,2,3), a dynamic equilibrium occurs, which mathematically can be written as:

(7) $$\frac{dz_{i}}{dt}+\frac{d_{m}B^{2}}{4\mu_{0}\eta_{i}}=0,$$

and represents the equation of motion for the CI microparticles in the dielectric component between the copper foils of the capacitors ${\mathrm{PEC}}_{i}$. At $t_{0}$, the distance between the dipoles **m** is $\delta_{i}$ (with i=1,2,3), and at a moment $t>t_{0}$, the distance between the same dipoles is $z_{i}<\delta_{i}$. With these conditions, we integrate Equation (7) and obtain:

(8) $$z_{i}=\delta_{i}\left(1-\frac{d_{m}B^{2}}{4\mu_{0}\eta_{i}\delta_{i}}t\right).$$

This formula describing the distance between dipoles in the capacitors ${\mathrm{PEC}}_{i}$ is derived under the assumption that the dipoles (i.e., CI microparticles) are subjected to a uniform magnetic field. The magnetic field induces dipole-dipole interactions, leading to the formation of chain-like structures aligned with the field direction. The distance between these dipoles decreases monotonically over time due to the attractive magnetic forces. This formula assumes a linear and uniform motion of the dipoles and is valid as long as the dipoles do not come into contact. In addition, the formula is valid for times *t* such that the term $3\pi d_{m}^{2}B^{2}/4\mu_{0}\eta_{i}\delta_{i}t$ remains less than 1, ensuring that $z_{i}$ remains positive and physically meaningful. The magnetic field should be strong enough to induce dipole formation but not so strong as to cause immediate aggregation of the particles. The suspension should have a viscosity $\eta_{i}$ that allows for measurable changes in $z_{i}$ over the experimental time frame. The paper by Dominguez-Garcia et al. (Ref. [40]) provides a detailed experimental investigation of the aggregation dynamics in magnetorheological fluids. The obtained results support the notion that the distance between magnetic particles in magnetorheological fluids decreases with time as they aggregate under a constant magnetic field, aligning with the theoretical framework described by Equation (8).

Between two dipoles **m** in each chain, a microcapacitor is formed. The electric capacitance $C_{z_{i}}$ (i=1,2,3) of a microcapacitor is approximated by the relation:

(9) $$C_{z_{i}}=\frac{\varepsilon_{0}\varepsilon_{i}^{\prime }S}{z_{i}},$$

where $\varepsilon_{0}$ is the dielectric constant of the vacuum, $\varepsilon_{i}^{\prime }$ is the relative dielectric permittivity of the ${\mathrm{MRS}}_{i}$ suspensions, *S* is the surface area of the dipoles **m**, and $z_{i}$ is the distance between the centers of mass of the dipoles in each chain. For $S=\pi d_{m}^{2}/4$ and the expression for $z_{i}$ (i=1,2,3) in Equation (8) inserted in Equation (9), we obtain the expression for the capacitance of a microcapacitor:

(10) $$C_{z_{i}}=\frac{0.25\varepsilon_{0}\varepsilon_{i}^{\prime }\pi d_{m}^{2}}{\delta_{i}\left(1-\frac{d_{m}B^{2}}{4\mu_{0}\eta_{i}\delta_{i}}t\right)}.$$

The maximum number $n_{1}$ of dipoles **m** in each chain is defined by the expression [14]:

(11) $$n_{1}=\frac{h_{0}}{d_{m}},$$

where $h_{0}$ is the thickness of the ${\mathrm{MRS}}_{i}$ suspensions. The capacitors $C_{z_{i}}$ (with i=1,2,3) are in series. Therefore, the equivalent electrical capacitance of a chain of dipoles is:

(12) $$C_{z_{{\mathrm{ch}}_{i}}}=\frac{C_{z_{i}}}{n_{1}-1}=\frac{0.25\varepsilon_{0}\varepsilon_{i}^{\prime }\pi d_{m}^{3}}{\delta_{i}h_{0}\left(1-\frac{d_{m}B^{2}}{4\mu_{0}\eta_{i}\delta_{i}}t\right)},\;\;\;\mathrm{for}\;\;\;n_{1}\gg 1$$

The number *N* of dipoles **m** in the volume of the ${\mathrm{MRS}}_{i}$ is estimated with the expression [14]:

(13) $$N=\frac{\Phi_{\mathrm{CI}}V}{V_{\mathrm{CI}}},$$

where *V* is the volume of the ${\mathrm{MRS}}_{i}$, and $V_{\mathrm{CI}}$ is the volume of a CI microparticle. For $V=\pi D^{2}h_{0}/4$ and $V_{\mathrm{CI}}=\pi d_{m}^{3}/6$ introduced in Equation (13), the expression for calculating the number *N* is obtained as follows:

(14) $$N=\frac{3D^{2}h_{0}}{2d_{m}^{3}}\Phi_{\mathrm{CI}},$$

where *D* is the diameter of the body formed by the ${\mathrm{MRS}}_{i}$.

The number of chains of magnetic dipoles is $n_{2}=N/n_{1}$. Using the expression for *N* given by Equation (14) and the value of $n_{1}$, we obtain the expression for calculating the number of chains of dipoles **m** in ${\mathrm{MRS}}_{i}$ as follows:

(15) $$n_{2}=\frac{3D^{2}\Phi_{\mathrm{CI}}}{2d_{m}^{2}}.$$

The capacitor chains are electrically connected in parallel through the copper foils. Therefore, the electrical capacitance of the capacitors ${\mathrm{PEC}}_{i}$ can be estimated using the relation $C_{i}=n_{2}C_{z_{{\mathrm{ch}}_{i}}}$. By introducing $n_{2}$ from Equation (15) and the value of $C_{z_{{\mathrm{ch}}_{i}}}$ from Equation (12), we obtain the relation for the capacitance of the capacitors ${\mathrm{PEC}}_{i}$ in a magnetic field, as:

(16) $$C_{i}=\frac{C_{0_{i}}}{1-\frac{d_{m}B^{2}}{4\mu_{0}\eta_{i}\delta_{i}}t}.$$

The value $C_{0_{i}}$ is the capacitance at the initial moment $t_{0}=0$ s of the capacitors ${\mathrm{PEC}}_{i}$ and has the form:

(17) $$C_{0_{i}}=\frac{0.75\pi \varepsilon_{0}\varepsilon_{i}^{\prime }D^{2}d_{m}\Phi_{\mathrm{CI}}}{2h_{0}\delta_{i}}.$$

It is observed from Equation (16) that the value $C_{i}$ depends on the geometric dimensions of the PECs, the diameter $d_{m}$, the volume fraction of the CI microparticles in the liquid matrix, and the volume fraction of the GP microparticles. By using numerical values D=20mm, $h_{0}=2\;\mathrm{mm}$, $d_{m}=5\;\mu m$, $\mu_{0}=4\pi \times 10^{-7}$ H/m, and the values $\delta_{i}$ with i=1,2,3 from Equations (1) and (2) we obtain:

(18) $$C_{1}=\frac{C_{0_{1}}}{1-\frac{2.18\times 10^{-5}\;B^{2}\left(\mathrm{mT}\right)t\left(s\right)}{\eta_{1}}},\;\mathrm{for}\;\mathrm{capacitor}\;{\mathrm{PEC}}_{1},$$

(19) $$C_{2}=\frac{C_{0_{2}}}{1-\frac{2.11\times 10^{-5}\;B^{2}\left(\mathrm{mT}\right)t\left(s\right)}{\eta_{2}}},\;\mathrm{for}\;\mathrm{capacitor}\;{\mathrm{PEC}}_{2},$$

and respectively,

(20) $$C_{3}=\frac{C_{0_{3}}}{1-\frac{2.05\times 10^{-5}\;B^{2}\left(\mathrm{mT}\right)t\left(s\right)}{\eta_{3}}},\;\mathrm{for}\;\mathrm{capacitor}\;{\mathrm{PEC}}_{3}.$$

## 6. Results

### 6.1. Stability of PECs with Lard, GP and Respectively CI Microparticles

**Figure 7:**
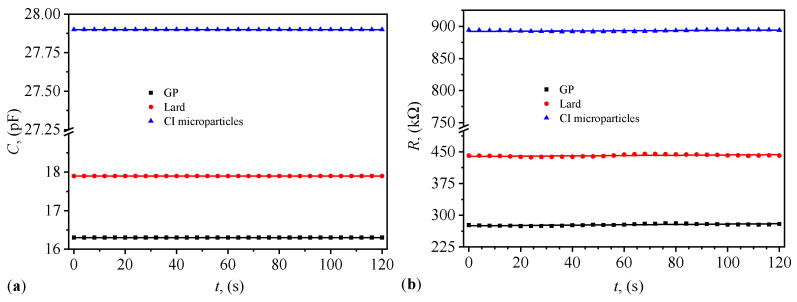
Variation of the equivalent electrical capacitance (**a**) and resistance (**b**) with time *t* for the electrical capacitors with lard, GP, and respectively CI microparticles.

The time dependence of the equivalent electrical capacitance *C* and resistance *R* for PECs with lard, GP, and respectively CI microparticles are shown in Figure 7a,b. The results show that both *C* and *R* depend on the type of the dielectric material used in PEC. Their behavior is quasi-constant with time *t* and thus *C* and *R* are stable during measurements.

The equivalent electrical resistance values *R* from Figure 7b and implicitly the average equivalent electrical resistances $R_{m}$ are the effect of contact resistances between CI microparticles. This phenomenon is confirmed in Refs. [41,42] for the case of microparticles composed of polypyrrole nanotubes decorated with magnetite nanoparticles and in Ref. [43] for the case of nickel microparticles coated with polypyrrole. These studies show that increasing the compression voltage decreases the resistance of the body formed by the microparticles. On the other hand, the electrical capacitance *C* and the average capacitance $C_{m}$ result from the formation of series and parallel microcapacitors [14] in the space occupied by the CI microparticles. The electrical conduction of lard is due to the presence of fatty acids (palmitic acid, stearic acid, oleic acid, and linoleic acid) and triglycerides [25]. The ratio of these components affects their dielectric properties [44]. The electrical conduction of the body formed by GP is due to contact resistance between the particles. Conversely, the intrinsic electrical conduction of GP and their dielectric properties is due to the presence of amino acids [45].

### 6.2. Electrical Properties of PECs

**Figure 8:**
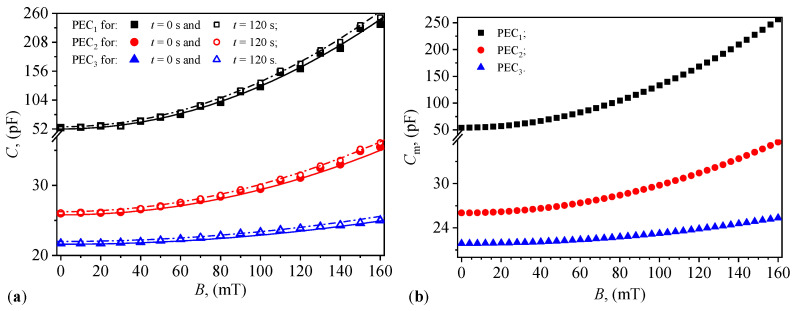
(**a**) The electrical capacitance *C* of capacitors ${\mathrm{PEC}}_{i}$ (i=1,2, and 3) as a function of *B* values of the magnetic flux density (points—experimental data; lines—polynomial fits; see Table 3 in Section 6.3 for details). (**b**) The corresponding average electrical capacitance $C_{m}$ of the same capacitors.

For PECs with MRS as dielectric material, the recorded data are graphically represented in Figure 8a. The average values, $C_{m}$, of the capacitance are shown in Figure 8b. These are obtained from the capacitance values recorded at t=0 s and t=120 s, corresponding to the *B* values of the magnetic flux density in Figure 8a. The experimental points are well approximated by the Equation (21) in Section 6.3.

From Figure 8a,b, it is observed that the values of $C_{i}$ and of $C_{m}$ for capacitors ${\mathrm{PEC}}_{i}$ (i=1,2, and 3) depend on the presence of the magnetic field and the presence of GP. In the absence of a magnetic field, the capacitances at B=0 mT depend on the volume fraction GP. They decrease by about half for the capacitor with $\Phi_{\mathrm{GP}}=\Phi_{\mathrm{CI}}$ (i.e., ${\mathrm{PEC}}_{2}$) and by about 2.5 times for the capacitor with $\Phi_{\mathrm{GP}}=2\Phi_{\mathrm{CI}}$ (i.e., ${\mathrm{PEC}}_{3}$). These results are in agreement with Equations (16) and (21). This equation, corroborated with Equation (2) shows that increasing the values of the distance $\delta_{i}$ (for i=1,2, and 3) between the mass centers of the CI microparticles results in a decrease in the values of $C_{0_{i}}$, in agreement with the experimental data in Figure 8. Note in Figure 8a that the capacitance of ${\mathrm{PEC}}_{1}$ is significantly greater as compared to any of its components, as shown in Figure 7a. This behavior can be explained by the interfacial polarization effect, synergistic interactions between the components, enhanced polarizability due to CI microparticles, and the optimal microstructural arrangement of the composite. These factors collectively contribute to the superior capacitance observed in ${\mathrm{PEC}}_{1}$.

### 6.3. Theoretical Models and Fitting Procedures for Capacitance Data

The experimental data in Figure 8a are fitted by polynomials of the form:

(21) $$C_{i}=C_{0_{i}}\left(1+\theta_{i}\cdot B^{2}\right),\;\mathrm{with}\;i=1,2,\;\mathrm{and}\;3,$$

in which $C_{i}$ and $C_{0_{i}}$ are the electrical capacitances of the capacitors ${\mathrm{PEC}}_{i}$ in the presence and absence of a magnetic field with magnetic flux density *B*, and $\theta_{i}$ is a dimensionless parameter whose magnitude depends on the composition of ${\mathrm{MRS}}_{i}$. The values of $C_{0_{i}}$ and $\theta_{i}$ corresponding to the capacitors ${\mathrm{PEC}}_{i}$ are listed in Table 3 for t=0s, and respectively for t=120s. Due to very small errors, the average values of the capacitance $C_{m}$ essentially coincide with $C_{0}$.

From an electrical point of view, PECs consist of a planar capacitor $C_{m}$ connected in parallel with a linear resistor $R_{m}$. Given the formula for calculating the electric capacity of a planar capacitor and, respectively, the formula of a linear resistor, we obtain the relative dielectric permittivity $\varepsilon_{r}^{\prime }$ and the dielectric loss coefficient $\varepsilon_{r}^{\prime \prime }$ of the dielectric materials between the electrodes of the PECs, as follows:

(22) $$\varepsilon_{r}^{\prime }=C_{m}h_{0}/\left(0.25\varepsilon_{0}D^{2}\right),$$

and respectively

(23) $$\varepsilon_{r}^{\prime \prime }=h_{0}/\left(0.5\pi \varepsilon_{0}fD^{2}R_{m}\right),$$

where *D* and $h_{0}$ are the diameter and thickness of the dielectric materials in the PEC capacitors; $\varepsilon_{0}$ is the vacuum permittivity constant; and *f* is the frequency of the alternating electric field. For $\varepsilon_{0}=8.854\;\mathrm{pF}/m$; f=1kHz; D=20mm; and $h_{0}=2\;\mathrm{mm}$ substituted in Equations (22) and (23), we obtain:

(24) $$\varepsilon_{r}^{\prime }=1.41\cdot C_{m}\;\left(\mathrm{pF}\right),$$

and respectively:

(25) $$\varepsilon_{r}^{\prime \prime }=0.22455/R_{m}\;(k\Omega ).$$

### 6.4. Rheological Properties of MRSs and Relative Dielectric Permittivity

In the presence of a magnetic field, the values of $C_{i}$ (for i=1,2, and 3) increase significantly with the increase in the values of *B* of the magnetic flux density, in agreement with Equations (16) and (21). This effect is due to the fact that during the time *t* of applying the value *B* of the magnetic flux density, the ratio $3\pi d_{m}^{2}B^{2}t/\left(4\mu_{0}\eta_{i}\delta_{i}\right)$ is always subunitary and remains constant. This is possible by increasing the value of $\eta_{i}$ of the viscosity of ${\mathrm{MRS}}_{i}$ with the increase in the value of *B* of the magnetic flux density, as will be shown later. The calculation relation of the viscosity $\eta_{i}$ of the suspensions ${\mathrm{MRS}}_{i}$ in the magnetic field is obtained from Section 5.

Thus, from Equations (18)–(20), where we set t=120s, we obtain the viscosity expressions for the MRS suspensions, namely:

(26) $$\eta_{1}\approx 11.05\cdot B^{2}\;\left(\mathrm{mT}\right)/\left(1-C_{0_{1}}/C_{1}\right),\;\mathrm{for}\;{\mathrm{MRS}}_{1},$$

(27) $$\eta_{2}\approx 10.70\cdot B^{2}\;\left(\mathrm{mT}\right)/\left(1-C_{0_{2}}/C_{2}\right),\;\mathrm{for}\;{\mathrm{MRS}}_{2},$$

(28) $$\eta_{3}\approx 10.39\cdot B^{2}\;\left(\mathrm{mT}\right)/\left(1-C_{0_{3}}/C_{3}\right),\;\mathrm{for}\;{\mathrm{MRS}}_{3}.$$

**Figure 9:**
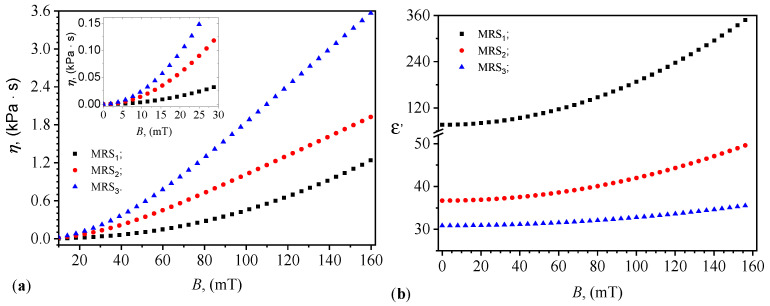
The viscosity η (**a**) and relative dielectric permittivity $\epsilon^{\prime }$ (**b**) of the suspensions ${\mathrm{MRS}}_{i}$ (with i=1,2,and3) as a function of the magnetic flux density *B*.

The functions $C_{i}=C_{i}{\left(B\right)}_{{\mathrm{PEC}}_{i}}$ from Figure 8, corresponding to i=1,2 and 3, are introduced in Equations (26), (27) and respectively in (28). At the end of this step, in Figure 9a, we obtain the functions $\eta_{i}=\eta_{i}{\left(B\right)}_{{\mathrm{MRS}}_{i}}$ (for i=1,2,and3). It can be observed from Figure 9a that the viscosity of the suspensions in a magnetic field is significantly influenced by the magnetic field, similar to the case of classical MRSs [38] and in agreement with the model developed in Section 5 (Equations (18)–(20)). From the same figure, it is also noted that for the same values of *B*, the viscosity η is influenced by the volume fraction of GP. By considering η as the coupling factor between shear stress and shear rate, the results obtained in Figure 9a are similar to those obtained in Ref. [38], where in a hybrid MRS the coupling coefficient between cotton microfibers increases with the increase in *B* and the amount of CI microparticles.

The measurement of viscosity in our MRSs in Figure 9a does not involve a conventional shear rate as typically defined in rheological studies. In traditional rheology, the shear rate is defined as the rate at which adjacent layers of fluid move relative to each other. However, our experimental setup and the behavior of the suspension under the influence of a magnetic field necessitate a different approach. The CI microparticles in our suspensions exhibit linear and uniform motion when subjected to a magnetic field. This motion aligns the magnetic dipoles formed by the microparticles along the direction of the magnetic flux density. Due to this uniform linear movement, the concept of shear (which involves relative motion between fluid layers) is not applicable in the traditional sense. Instead of shear, we describe the interaction between the magnetic dipoles and the base liquid in terms of a coupling coefficient. This coefficient reflects the influence of the magnetic field on the viscosity of the suspension. The coupling coefficient is a function of the magnetic flux density and characterizes the resistance to the motion of the magnetic dipoles within the suspension. In Figure 9a, the plotted viscosity values are derived from the magnetic interactions and the resulting coupling between the magnetic dipoles and the base liquid. This viscosity represents the effective resistance to the uniform linear motion of the microparticles under the applied magnetic field, rather than a traditional shear-induced viscosity. Therefore, while a conventional shear rate is not defined in our experiments, the viscosity values presented in Figure 9a accurately reflect the dynamic behavior of the MRSs under the influence of a magnetic field. The use of a coupling coefficient instead of a shear rate provides a more appropriate description of the system’s response to the applied magnetic flux density.

The results shown in Figure 9a are also similar to those obtained in Ref. [37], where in hybrid MRS, the coupling coefficient between the cotton microfibers increases with the size of the magnetic field and the amount of CI microparticles. As pointed out above, when a magnetic field is applied, magnetic interactions are established between the magnetic dipoles (CI microparticles) within the suspension. These interactions are highly sensitive to the magnetic flux density *B*. According to Equation (8), the magnetic dipoles move closer to each other under the influence of the magnetic field. The magnetic dipoles tend to form aggregates, often in the form of chains, as shown in the optical microscopy in Figure 1d or as depicted in the model in Figure 6. As these dipoles aggregate, they interact with other dipoles, generating shear stresses within the suspension. These shear stresses increase with *B* and the volume fraction of GP in the suspension $\Phi_{\mathrm{GP}}$. The shear stresses generated by the magnetic interactions and the movement of dipoles give rise to shear bends in the suspension. The coupling coefficient between these shear stresses and the shear speeds (which define the viscosity) increases with *B* and $\Phi_{\mathrm{GP}}$.

The relative dielectric permittivity is calculated using Equation (22). In this expression, we introduce the functions $C_{m}=C_{m}{\left(B\right)}_{{\mathrm{PEC}}_{i}}$, for i=1,2,and3, from Figure 8b, and we obtain in Figure 9b the functions $\epsilon^{\prime }=\epsilon^{\prime }{\left(B\right)}_{{\mathrm{MRS}}_{i}}$. It can be observed from this figure that the values of $\epsilon^{\prime }$ for the MRSs increase significantly with the increase in magnetic flux density, similar to classical MRSs [32]. However, the GP creates layers within the compositional structure of the ${\mathrm{MRS}}_{i}$, for i=2,3 suspensions between the lines of magnetic dipoles (CI microparticles). The resulting effect is the creation of capacitors connected in series between the copper foils of the ${\mathrm{PEC}}_{i}$ in the absence and presence of the magnetic field. The distance between the plates of these capacitors increases with the increase in the value of $\Phi_{\mathrm{GP}}$, as suggested by the results in Figure 8a,b.

This type of dependence is specific to composites based on magnetorheological suspensions. Thus, in Ref. [14], an MRS suspension is manufactured using honey with CI microparticles. The obtained suspension is used to impregnate a commercial cotton fabric fitted with two copper electrodes. The assembled structure is introduced into molten beeswax. After cooling, a planar capacitor is obtained, with the distance between the plates being 10 mm. In a static magnetic field superimposed on an alternating electric field with a frequency of f=1 kHz, using the planar capacitor method developed in Ref. [46], the equivalent dielectric permittivity is obtained from equivalent electrical capacitance measurements. Thus, at B=0.0 T, $\epsilon^{\prime }=2\times 10^{4}$, while for B=0.2 T, $\epsilon^{\prime }$ increases by about one order of magnitude. By using the same planar capacitor method, the relative dielectric permittivity of smart tissues prepared by impregnation of an absorbent cloth with SO mixed with CI and various volume concentrations of $\gamma -{\mathrm{Fe}}_{3}O_{3}$ nanoparticles is determined. It is shown that the size increases with the increase in the *B* value of the magnetic flux density. Thus, in the absence of microfibers from iron oxides, $\epsilon^{\prime }=7000$ at B=0 mT. The values of the relative dielectric permittivity increase with the increase of the B value of the magnetic flux density, such that at B=320 mT, $\epsilon^{\prime }$ = 19,000.

**Figure 10:**
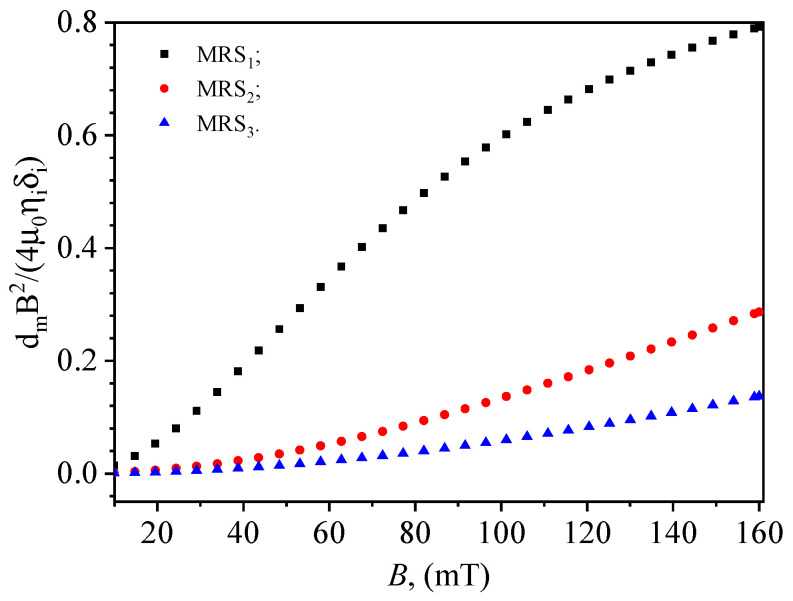
Variation with magnetic flux density *B* of the second term in the denominator of Equation (16).

By using numerical values t=120 s, $\delta_{i}$ given by Equations (1) and (2), and the variation of η from Figure 9a, the second term in the denominator of Equation (16) depends only on the magnetic flux density. Its behavior, for the three MRSs is shown in Figure 10. One can see that for ${\mathrm{MRS}}_{2}$ and ${\mathrm{MRS}}_{3}$ in the whole range of *B*, and for B≲50 mT in the case of ${\mathrm{MRS}}_{1}$, this term is much smaller than 1. Thus, by performing a Taylor series expansion around zero and truncating it to the first two terms, Equation (16) can be rewritten as:

(29) $$C_{i}=C_{0_{i}}\left(1+\frac{d_{m}B^{2}}{4\mu_{0}\eta_{i}\delta_{i}}t\right).$$

### 6.5. Magnetodielectric Effects in MRSs

We define the magnetodielectric effect using the expression:

(30) $${\mathrm{MDE}}_{i}\left(\%\right)={\left(\frac{C_{m}}{C_{m_{0}}}-1\right)}_{{\mathrm{PEC}}_{i}}\times 100,\;\mathrm{for}\;i=1,2,3.$$

**Figure 11:**
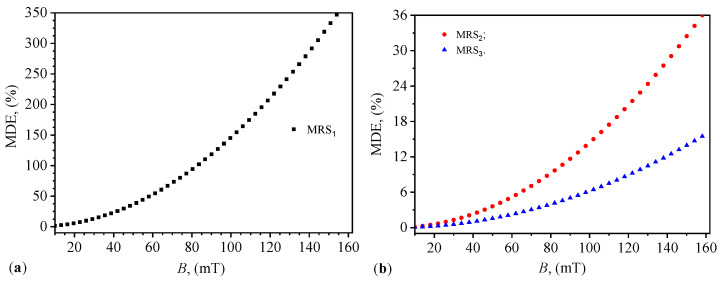
Magnetodielectric effect MDE in ${\mathrm{MRS}}_{1}$ (**a**), and in ${\mathrm{MRS}}_{2}$ and ${\mathrm{MRS}}_{3}$ (**b**) as a function of the magnetic flux density *B*.

The functions $C_{m}=C_{m}{\left(B\right)}_{{\mathrm{PEC}}_{i}}$ for i=1,2,3. Figure 8b are substituted into Equation (30) yielding the functions $\mathrm{MDE}=\mathrm{MDE}{\left(B\right)}_{{\mathrm{MRS}}_{i}}$ as shown in Figure 11. The results show that the magnetodielectric effect of the ${\mathrm{MRS}}_{i}$ suspensions (for i=1,2,3) is significantly influenced by the magnitude of the *B* magnetic flux density. This effect is also seen in classical MRS suspensions [14]. The introduction of gelatin decreases the MDE magnitude as the $\Phi_{\mathrm{GP}}$ value increases. For $\Phi_{\mathrm{GP}}=10\;\mathrm{vol}\%$ at B=100mT, the MDE is approximately 14.39. However, at the same *B* value, the MDE magnitude decreases by approximately 2.3 times for ${\mathrm{MRS}}_{3}$ (see Figure 11b). This effect is due to the increased initial distance between CI microparticles as a result of the increased $\Phi_{\mathrm{GP}}$ value (see Equation (1) in conjunction with Equations (16) and (17)).

## 7. Discussion

The results obtained from the experimental investigation of MRSs composed of lard, GP, and CI microparticles have demonstrated several noteworthy findings. These findings contribute to the broader context of existing literature on MRSs and their applications.

The stability of PECs using these MRSs was confirmed through time-dependent measurements of capacitance *C* and resistance *R* (Figure 7). The quasi-constant behavior of these properties over time indicates that the suspensions maintain their performance characteristics under operational conditions, which is critical for practical applications in electronics and other industries.

The dynamic viscosity η (Figure 9a) and relative dielectric permittivity $\epsilon^{\prime }$ (Figure 9b) of the suspensions were found to be dependent on both *B* and $\Phi_{\mathrm{GP}}$. As *B* increased, both η and $\epsilon^{\prime }$ exhibited significant increases, a phenomenon similarly observed in traditional MRSs. The presence of GP, however, introduced an additional layer of complexity, acting as dielectric barriers and modifying the capacitance and resistance within the suspensions. This dual role of GP as both a structural and functional component underscores its importance in fine-tuning the properties of MRSs.

The magnetodielectric effect MDE (Figure 11) observed in the suspensions also varied significantly with *B* and $\Phi_{\mathrm{GP}}$. Specifically, the MDE was shown to decrease with increasing $\Phi_{\mathrm{GP}}$, which is attributed to the increased initial distance between CI microparticles, thereby reducing magnetic interactions. This is consistent with the theoretical models based on the dipolar approximation and previous studies that highlight the role of particle distribution in MRS behavior [20].

The implications of this study are manifold, suggesting several avenues for future research. Further refinement of the ratios and types of biodegradable materials could enhance the performance and stability of MRSs, making them suitable for a wider range of applications. Investigating the long-term stability and performance of these suspensions under varying environmental conditions would provide deeper insights into their practical viability. Exploring the use of these MRSs in advanced technological applications, such as smart materials for adaptive systems or in medical devices, could open new frontiers for research and development. Enhancing the theoretical models to better predict the behavior of such complex suspensions under different operational scenarios could lead to more accurate and reliable designs of MRS-based devices.

These findings extend the work of prior research in several key areas. Previous studies have explored various biodegradable and renewable materials for MRSs, such as nanocellulose [1] and honey [13]. The use of lard and gelatin in this study adds to the growing body of literature on sustainable alternatives, emphasizing the potential for low-cost and environmentally friendly MRSs. The observed MDEs are consistent with those reported in MRSs based on magnetorheological suspensions [4]. The ability to achieve similar effects with other sustainable materials highlights the versatility and adaptability of the developed suspensions. Controlling the viscosity of MRSs is crucial for their application in devices like dampers and clutches. The results (Figure 9a) align with studies that have shown the impact of magnetic fields on viscosity, further validating the use of MRSs in mechanical and automotive applications [18,19].

An important issue for such MRSs is the long-term stability (over several months or years) of lard and GP. Lard, being a natural fat, is susceptible to oxidative rancidity when exposed to air over extended periods [24]. This oxidative process can result in the formation of off-flavors and odors due to the breakdown of fat molecules into peroxides and secondary oxidation products. However, the rate of this degradation can be minimized through proper storage conditions, such as storage in airtight containers (limiting exposure to air reduces the availability of oxygen, which is necessary for the oxidation process), cool storage conditions (storing lard in a cool environment slows down the oxidation reaction kinetics), and the addition of antioxidants (natural antioxidants, such as tocopherols, can be added to lard to inhibit oxidative reactions and extend its shelf life). Gelatin, a protein derived from collagen, is generally more chemically stable than fats [47]. However, it can also undergo changes over time when exposed to air and varying humidity levels. The primary concerns with gelatin stability include moisture absorption (gelatin is hygroscopic and can absorb moisture from the air, which may lead to swelling or dissolution) and microbial growth (if exposed to high humidity or moisture, gelatin can be susceptible to microbial contamination). Thus, by taking into account that the MRSs are well compacted within the ST tape (as shown in Figure 3), the exposure to air and moisture is minimized, so we can ensure long-term stability and performance of these natural components in our MRSs for at least a few months.

## 8. Conclusions

This study successfully demonstrates the preparation and characterization of MRSs using lard, GP, and CI microparticles. The findings indicate that these low-cost and eco-friendly materials can effectively replace traditional synthetic materials in MRSs, offering similar magnetodielectric and rheological properties. The constructed cylindrical capacitors showed significant increases in dynamic viscosity and relative dielectric permittivity with increasing magnetic flux density and decreases, with increasing gelatin volume fraction. These effects are consistent with those observed in conventional MRSs, suggesting that the newly developed suspensions can be viable substitutes in various applications.

The experimental results highlight the critical role of the magnetic field in influencing the properties of the suspensions, validating their potential use in mechanical and automotive applications where viscosity control is essential. The study also underscores the dual role of gelatin as both a structural and functional component, enhancing the fine-tuning capabilities of MRS properties. The observed magnetodielectric effect and its dependence on the volume fraction of gelatin and magnetic flux density align with theoretical models, providing a robust foundation for further research and development.

Overall, this study contributes significantly to the field of magnetorheological materials, presenting a sustainable alternative that aligns with the principles of circular economy and environmental stewardship. The promising results pave the way for future innovations and applications in various industrial and technological domains.

## Figures and Tables

**Table 1:** Mass densities (ρ), relative dielectric permittivity ($\varepsilon_{r}^{\prime }$), dielectric loss factor ($\varepsilon_{r}^{\prime \prime }$) and the loss tangent (*D*) for lard, GP and CI microparticles.

	ρ (g/${\mathrm{cm}}^{3}$)	$\varepsilon_{r}^{\prime }$	$\varepsilon_{r}^{\prime \prime }$ (×$10^{-4}$)	*D* ($\times 10^{-5}$)
Lard	0.8845	25.1483	8.09291	3.21807
GP	0.9200	22.9860	5.091925	2.21523
CI	7.8600	39.339	2.515722	0.63949

**Table 2:** Volumes *V* and volume fractions Φ of the MRS components.

	$V_{\mathrm{lard}}$ (${\mathrm{cm}}^{3}$)	$V_{\mathrm{CI}}$ (${\mathrm{cm}}^{3}$)	$V_{\mathrm{GP}}$ (${\mathrm{cm}}^{3}$)	$\Phi_{\mathrm{lard}}$ (vol.%)	$\Phi_{\mathrm{CI}}$ (vol.%)	$\Phi_{\mathrm{GP}}$ (vol.%)
${\mathrm{MRS}}_{1}$	3.6	0.4	0.0	90	10	0
${\mathrm{MRS}}_{2}$	3.2	0.4	0.4	80	10	10
${\mathrm{MRS}}_{3}$	2.8	0.4	0.8	70	10	20

**Table 3:** Values of the parameters $C_{0_{i}}$, $\theta_{i}$ obtained by fitting data in Figure 8 with Equation (21), at time t=0 s and t=120 s.

	$C_{0_{i}}$ (pF) at t=0 s	$\theta_{i}$ (pF/${\mathrm{mT}}^{2}$) at t=0 s	$C_{0_{i}}$ (pF) at t=120 s	$\theta_{i}$ (pF/${\mathrm{mT}}^{2}$) at t=120 s
${\mathrm{PEC}}_{1}$	52	1.4808 × $10^{-4}$	56	1.4464 × $10^{-4}$
${\mathrm{PEC}}_{2}$	25.8	1.3953 × $10^{-5}$	26.25	1.4857 × $10^{-5}$
${\mathrm{PEC}}_{3}$	21.8	5.9698 × $10^{-6}$	222	6.3636 × $10^{-6}$

**Figure A1:**
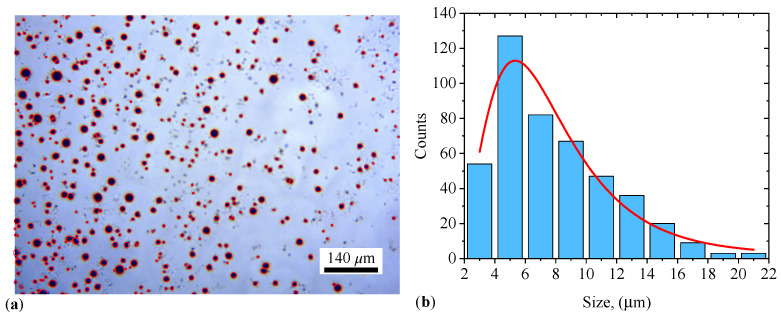
(**a**) Photo of GPs as shown in Figure 1a but with marked particles (red disks) for which the size distribution has been calculated. (**b**) The corresponding size distribution and a fit (red curve) with a log-normal function.

## Data Availability

The original contributions presented in the study are included in the article, further inquiries can be directed to the corresponding author.

## Appendix A. Size Distribution of GPs

The average diameter of GP is 6.94 ± 0.55 μm and it has been determined from a set of 448 particles, marked in red circles in Figure A1a. To this aim a log-normal fit has been used on the size distribution (see Figure A1b).

## Appendix B. PECs with Lard, CP and GP Microparticles

The lard, gelatin and CI microparticles are introduced into measurement cells, as shown in Figure A2. By consolidating with adhesive tape, capacitors of the type shown in Figure 3 are obtained.
